# Antimicrobial Activity of Medicinal Plants Correlates with the Proportion of Antagonistic Endophytes

**DOI:** 10.3389/fmicb.2017.00199

**Published:** 2017-02-09

**Authors:** Dilfuza Egamberdieva, Stephan Wirth, Undine Behrendt, Parvaiz Ahmad, Gabriele Berg

**Affiliations:** ^1^Institute of Landscape Biogeochemistry, Leibniz Centre for Agricultural Landscape ResearchMüncheberg, Germany; ^2^Department of Botany and Microbiology, Faculty of Science, King Saud UniversityRiyadh, Saudi Arabia; ^3^Department of Botany, Sri Pratap CollegeSrinagar, India; ^4^Institute of Environmental Biotechnology, Graz University of TechnologyGraz, Austria

**Keywords:** *Hypericum perforatum*, *Ziziphora capitata*, endophytic bacteria, plant growth traits, antimicrobial activity, antagonism

## Abstract

Medicinal plants are known to harbor potential endophytic microbes, due to their bioactive compounds. In a first study of ongoing research, endophytic bacteria were isolated from two medicinal plants, *Hypericum perforatum* and *Ziziphora capitata* with contrasting antimicrobial activities from the Chatkal Biosphere Reserve of Uzbekistan, and their plant-specific traits involved in biocontrol and plant growth promotion were evaluated. Plant extracts of *H. perforatum* exhibited a remarkable activity against bacterial and fungal pathogens, whereas extracts of *Z. capitata* did not exhibit any potential antimicrobial activity. Matrix-assisted laser desorption ionization (MALDI) time-of-flight (TOF) mass spectrometry (MS) was used to identify plant associated culturable endophytic bacteria. The isolated culturable endophytes associated with *H. perforatum* belong to eight genera (*Arthrobacter, Achromobacter, Bacillus, Enterobacter, Erwinia, Pseudomonas, Pantoea, Serratia*, and *Stenotrophomonas*). The endophytic isolates from *Z. capitata* also contain those genera except *Arthrobacter*, *Serratia*, and *Stenotrophomonas*. *H. perforatum* with antibacterial activity supported more bacteria with antagonistic activity, as compared to *Z. capitata*. The antagonistic isolates were able to control tomato root rot caused by *Fusarium oxysporum* and stimulated plant growth under greenhouse conditions and could thus be a cost-effective source for agro-based biological control agents.

## Introduction

Medicinal plants are traditionally used worldwide as remedies for the treatment of various diseases, including asthma, gastrointestinal symptoms, skin disorders, respiratory and urinary problems, and hepatic and cardiovascular disease (Van Wyk and Wink, 2004; Tian et al., 2014). These plants synthesize a diverse array of biologically active compounds (Bajguz, 2007; Cushnie et al., 2014) that are important for them to survive and flourish in the natural environment, including protective functions with respect to abiotic stresses derived from temperature, water status, mineral nutrient supply and to insect pests (Simmonds, 2003; Treutter, 2006; Vardhini and Anjum, 2015). The composition of biologically active compounds of medicinal plants varies widely depending on the plant species, soil type and on their association with microbes (Zhao et al., 2011; Morsy, 2014). These bioactive secondary metabolites synthesized by medicinal plants can also strongly affect plant-associated microbial communities and their physiological functions (Qi et al., 2012; Philippot et al., 2013; Chaparro et al., 2014; reviewed in Köberl et al., 2013). Moreover, plants rely on their microbiome for specific traits and activities, including growth promotion, nutrient acquisition, induced systemic resistance and tolerance to abiotic stress factors (Egamberdieva et al., 2010, 2011; Malfanova et al., 2011; Sessitsch et al., 2013; Berg et al., 2014). Although a vast number of medicinal plants have been well-studied with respect to their phytochemical constitutes and pharmacological properties, their microbiome and the physiological interactions between host and microbes remain poorly understood (Köberl et al., 2014).

The plant-associated microbiome consists of distinct microbial communities living in the roots, shoots and endosphere (Beneduzi et al., 2012; Berg et al., 2014). The rhizosphere of many plants is well-studied and known to be a potential source for selecting beneficial microbes that can positively affect plant health (Weller et al., 2002; Berendsen et al., 2012; Philippot et al., 2013). Hence, understanding the response of microbial communities to alterations in the physiochemical environment of the rhizosphere may provide valuable insights into the microbial ecology of plant-associated bacteria. Köberl et al. (2013) observed a high abundance of antagonistic bacteria in the rhizosphere of the medicinal plants *Matricaria chamomilla*, *Calendula officinalis*, and *Solanum distichum*. The root-associated bacteria of *Ajuga bracteosa* exhibited a wide range of plant growth promoting activities by producing siderophores and indole acetic acid and exhibiting antioxidant activity (Kumar et al., 2012). Recently, endophytic microorganisms have been under increased investigation due to their intimate interaction with the host (Hardoim et al., 2015); it is believed that the phytochemical constitutes of plants are related either directly or indirectly to endophytic microbes and their interactions with host plants (Chandra, 2012; Qi et al., 2012). Despite first studies of endophytes in medicinal plants (Bharti et al., 2012; López-Fuentes et al., 2012; Miller et al., 2012; El-Deeb et al., 2013; Egamberdieva and Teixeira da Silva, 2015), the potential of medicinal plants is far from exhausted.

Therefore, the current exploratory study was designed to evaluate whether medicinal plants with contrasting antimicrobial activities have an impact on plant-specific traits involved in biocontrol and plant growth promotion of root-associated culturable endophytic bacteria. In first experiments of ongoing research, we studied *Ziziphora capitata* L. (Field basil) and *Hypericum perforatum* L. (St John’s wort) from the Chatkal Biosphere Reserve of Uzbekistan, an isolated protected area in Western Tien Shan province, which significantly surpasses other areas with respect to the absolute number of endemic species (Kogure et al., 2004). *Z. capitata* L. is a medicinal and aromatic plant of the *Lamiaceae* family, which is traditionally used for the treatment of various ailments, such as heart disease, inflammation, depression, diarrhea, fever, skin disorders, hepatic diseases, and edema (Sonboli et al., 2006). The *Ziziphora* species are rich in essential oils, flavanoids and sterols (Zhaparkulova et al., 2015). The major component of essential oil found in several species of *Ziziphora* is pulegone, which has strong antibacterial and antifungal activity (Sonboli et al., 2006), but *Z. capitata* does not contain pulegone (Ebrahimi et al., 2009). *H. perforatum* is a species in the family *Hypericaceae* and is known for analgesic, sedative, antihelmintic, anti-inflammatory, and antibacterial properties (Dall’Agnol et al., 2003). *H. perforatum* contains a wide range of biological active compounds, such as essential oils, tannins, flavonoids, xanthones, and hyperforin as an antibiotic substance (Jurgenliemk and Nahrstedt, 2002). The crude extracts of *H. perforatum* exhibited higher antibacterial activity against Gram-positive than Gram-negative bacteria (Sarkisian et al., 2012). The aim of this study was to isolate and characterize endophytic bacteria from two medicinal plants, *H. perforatum* and *Z. capitata*, with contrasting antimicrobial activities and evaluate their plant-specific traits involved in biocontrol and plant growth promotion.

## Materials and Methods

### Collection of Plant Samples

*Hypericum perforatum* (*Hypericaceae*) and *Ziziphora capitata* (*Lamiaceae*) plants were collected during the summer (June 2013, the plant’s flowering stage) from Chatkal Biosphere Reserve of Uzbekistan, western part of Tien Shan mountain (41°08′ N; 69°59′ E). This biosphere reserve is situated in the Tashkent Region within the Chatkal mountain range (1.110–4.000 m above sea level) of the West Tien-Shan Mountains and is unique for its significant role in biodiversity conservation and ethnobotany. The climate is characterized by average annual temperatures ranging from 20 to 25°C with increased annual precipitation from plains to mountains, reaching 700–800 mm.

### Preparation of Plant Extracts

The aerial parts of *H. perforatum* and *Z. capitata* were dried in the laboratory excluding direct sun light at room temperature for 6–7 days and ground into a fine powder by mortar and pestle. Approximately, 10 g of plant powder was extracted with 50 ml of methanol for 24 h in a dark room temperature. Subsequently, the solvent was evaporated in a rotary vacuum evaporator at 40°C and re-suspended in dimethyl sulfoxide (DMSO). The homogenate was filtered through Whatman No. 1 filter paper, centrifuged at 5000 *g* for 15 min and sterilized by filtration through 0.22-μm sterile filters (Millipore, Bedford, MA, USA). The filtrates were stored at -4°C and used for *in vitro* screening of antimicrobial activity.

### Antimicrobial Activity of Plant Extracts

The extracts were individually tested against the following pathogenic microorganisms: *Klebsiella oxytoca* 6653, *K. pneumoniae* 40602, *K. aerogenes* NCTC 8172, *Citrobacter freundii* 82073, *Staphylococcus aureus* MRSA 16, *Enterococcus faecalis* NCTC 775, *Providencia rettgeri* NCIMB 9570, *Pseudomonas aeruginosa* NCTC 6749, *Escherichia coli* NCTC 9001 and *Fusarium solani*, *Fusarium oxysporum*, and *Alternaria alternata*. Reference strains and clinical isolates were obtained from the Department of Microbiology, Manchester Metropolitan University, UK, and the National Culture Type Collection (NCTC), UK. The fungal strains were obtained from the Department of Microbiology and Biotechnology, National University of Uzbekistan. Each plant extract was dissolved in dimethyl sulfoxide (DMSO), sterilized by filtration using a sintered glass filter, and stored at 4°C. The antimicrobial activity of the extracts was tested using the agar well-diffusion method. Microorganisms were grown overnight at 30°C in Mueller-Hinton Broth (Oxoid, Basingstoke, UK) supplemented with 5% horse blood, and 100 μl of suspension containing 10^6^ CFU ml^-1^ of bacteria was spread on the surface of Mueller-Hinton agar plates. Wells with 6-mm diameters were cut off and filled with 50 μL of each extract (10 mg ml^-1^). Ampicillin (Sigma-Aldrich, Steinheim, Germany) (0.5 mg ml^-1^), nystatin (Sigma-Aldrich, Steinheim, Germany) (1 mg ml^-1^) and DMSO were used as controls. Fungal strains were grown on potato dextrose agar plates (PDA; Difco Laboratories, Detroit, MI, USA) at 28°C for 5 days. Small piece of fungal culture were placed in the middle of Petri plates. Each antimicrobial assay was performed in triplicate. The plates were incubated at an appropriate growth temperature for 2 days for bacterial strains (37°C) and 4 days for fungal strains (30°C). The assessment of antimicrobial activity was based on the measurement of inhibition zones on the agar surface around the well.

### Isolation of Endophytic Bacteria

Three plants from each species of *H. perforatum* and *Z. capitata* including roots (20–30 cm depth) were randomly collected about 1 m apart from each other from an area of 100 m^2^ in the Chatkal Biosphere Reserve. The whole plants, along with root systems, were wrapped in plastic bags, and brought to the laboratory on same day and immediately stored at 4°C. The isolation of bacterial strains was carried out on the next day to minimize storage effects.

The root systems of the collected plants were separated from the shoots, soil adhering to the roots was removed and roots were carefully washed under running water, taking care to minimize root injury. Three plants of each species were used to determine the number of bacterial colonies cultured from the root tissue. For the bacterial isolation, root tissues were pooled from each of three replicate plants. The roots were surface sterilized by immersion in 70% (v/v) ethanol, following by shaking in 5% (w/v) sodium hypochlorite solution for 5 min. Subsequently, the roots were rinsed in sterile distilled water six times. To test the efficiency of sterilization, the sterile roots were incubated in TSA medium for 2 days at 28°C, and no infestation was observed.

Sterilized roots were weighed aseptically (1 g) and macerated in a mortar employing phosphate buffered saline (PBS) (20 mM sodium phosphate, 150 mM NaCl, pH 7.4) in a laminar air flow cabinet. The extracts were placed in a tube containing 9 ml sterile PBS and shaken with a vortex for 1 min. The supernatant was collected and serially diluted (10^1^–10^5^) in PBS, and 100 μl from appropriate dilutions were spread on Tryptic Soy Agar (TSA, Difco Laboratories, Detroit, MI, USA) plates in triplicate. The plates were incubated at 28°C, and colony forming units (cfu) g^-1^ root tissue were determined on the third day. A representative number of colonies that exhibited differentiable colony morphologies were picked from plates and were re-streaked for the purification of the isolates. The pure bacterial cultures were preserved on plates at 4°C for the further analyses. In addition all bacterial isolates were stored in Tryptic Soy broth (TSB) (Difco) with 30% glycerol at -80°C.

### Identification of Endophytic Bacterial Strains

The identification of bacterial isolates was performed by matrix-assisted laser desorption/ionization time of flight mass spectrometry (MALDI-TOF MS) as described previously (Egamberdieva et al., 2016). The sample preparation was performed according to the ethanol/formic acid extraction protocol recommended by Bruker Daltonics (Bremen, Germany) and was described in Egamberdieva et al. (2016). Briefly, the isolates were cultured on TSA medium (Difco Laboratories, Detroit, MI, USA) for 24 h, and approximately 10 mg of cell mass was suspended in 300 μL water (LC–MS CHOMASOLV®; Honeywell) and vortexed to generate a homogenous suspension. The suspension was mixed with 900 μL ethanol (≥99.8% GC; Sigma-Aldrich) and centrifuged. The pellet was resuspended in 50 μL 70% formic acid (v/v) and subsequently carefully mixed with 50 μL acetonitrile. After centrifugation, aliquots of 1 μL supernatant were placed immediately on spots of a MALDI target. Each spot was allowed to dry and subsequently overlaid with 1 μL of matrix (α-ciano-4-hydroxycinnamic acid in 50% aqueous acetonitrile containing 2.5% trifluoroacetic acid). Mass spectra were acquired using a MALDI-TOF MS spectrometer in a linear positive mode (Microflex^TM^LT, Bruker Daltonics, Bremen, Germany) in a mass range of 2–20 kDa. A bacterial test standard (BTS, Bruker Daltonics, Bremen, Germany) was used for instrument calibration. The raw spectra were imported into the MALDI Biotyper^TM^ software and then processed and analyzed using standard pattern matching against the reference spectra in the MALDI Biotyper^TM^ reference database (version 3.0, Bruker Daltonics, Bremen, Germany). A calculated matching score (score value) provided a measure of the probability of a correct classification.

### *In vitro* Screening for Plant Beneficial Traits

The production of IAA (indole 3-acetic acid) was determined as described by Bano and Musarrat (2003). The IAA concentration in culture was calculated using a calibration curve of pure IAA as a standard. The cellulose-degrading ability of bacterial isolates was analyzed by streaking inocula on cellulose (Sigma-Aldrich, St. Louis, MO, USA) Congo-Red agar media as described by Pratima et al. (2012). Furthermore, β-1,3 glucanase activity was tested using the substrate lichenan (Sigma-Aldrich, St. Louis, MO, USA) in top agar plates (Walsh et al., 1995), and protease activity was determined by using 5% skimmed milk agar plates (Brown and Foster, 1970). The production of HCN by bacterial isolates was measured using the protocol described by Castric (1975).

The bacterial isolates were tested *in vitro* for their antagonistic activities against the following pathogenic fungi: *Fusarium oxysporum* f.sp. *radicis-lycopersici* (Forl), *F. solani*, *F. culmorum, Gaeumannomyces graminis* pv. *tritici* (Ggt), *Alternaria alternata*, and *Botrytis cinerea* and the oomycete *Pythium ultimum.* The bacterial isolates were grown in TSB broth for 3 days, and 50-μl bacterial cultures were dropped into a hole of PDA plates (4 mm in diameter). Fungal strains for inoculation were grown in peptone dextrose agar (PDA) plates at 28°C for 5 days. Disks of fresh cultures of the fungus (5 mm diameter) were cut out and placed 2 cm away from the hole filled with bacterial filtrate. The plates were sealed with Parafilm^®^M and incubated at 28°C in darkness until the fungi had grown over the control plates without bacteria. Antifungal activity was recorded as the width of the zone of growth inhibition between the fungi and the bacteria tested.

### Biological Control of Tomato Root Rot

Bacterial isolates with antagonistic activity against the majority of tested fungal pathogens, were tested for their ability to control tomato root rot caused by *F. oxysporum* f.sp. *radicis-lycopersici*. For the inoculation of soil, *F. oxysporum* was grown in PDA plates for 5 days. Small pieces of agar from the growing edge of the colony were homogenized and used to inoculate 300 ml of Chapek-Dox medium, which was kept under aeration (110 rpm) at 28°C. After 3 days, the spore suspension was filtrated with sterile glass wool to remove the mycelium. The concentration of spores in the inoculum was adjusted to 10^7^ spores ml^-1^ by microscopic enumeration with a cell-counting haemocytometer and mixed thoroughly with potting soil to obtain a concentration of approximately 10^7^ spores kg^-1^ soil. The tomato seeds of the cultivar Fuji Pink (Sakata, Japan) were sterilized by stirring with 70% ethanol for 5 min and in household bleach (adjusted to approximately 5% sodium hypochlorite) for 3 min. Subsequently, the seeds were washed several times with sterile distilled water. After germination in sterile Petri plates, the seeds were placed in a bacterial suspension of 1 × 10^8^ CFU ml^-1^ prepared as described above and shaken gently for 10 min. The inoculated seeds were sown in plastic pots, and each treatment contained four groups of 24 plants. The plants were grown in a growth chamber under controlled conditions (16 h light, 8 h dark), at temperature light 28°C, dark 20°C and relative humidity 60%. After 3 weeks, the plants were removed from the soil, washed and examined for foot and root rot symptoms as indicated by browning and lesions. Roots without any disease symptoms were classified as healthy.

### Plant Growth Stimulation

To test whether bacterial isolates were capable of stimulating plant growth, a pot experiment was conducted in the controlled plant growth chamber. Tomato seeds (*Solanum lycopersicum.* cv. Fuji Pink, Sakata, Japan) were surface-sterilized as described above. Surface-sterilized seeds were transferred to plastic Petri dishes and germinated for 4 days in a dark room at 25°C. The bacterial isolates were grown overnight in TSB, and 1 ml of each culture was pelleted by centrifugation (10.000 × *g* for 10 min). Cell pellets were washed with 1 ml PBS, re-suspended in PBS and cell suspensions were adjusted to OD_620__nm_ = 0.1 (0.2 for *Bacillus* and *Arthrobacter*) that correspond to a cell density of about 10^7^–10^8^ cells ml^-1^. Germinated tomato seeds were placed in the bacterial suspension with a sterile forceps and shaken gently. After 10 min, the inoculated seeds were aseptically planted into a plastic pot filled with potting soil (N 250 mg l^-1^, P 120 mg l^-1^, K 700 mg l^-1^, pH 6.0, Floragard GmbH, Germany) to a depth of approximately 1.5 cm. Non-inoculated plants were used as negative controls. Each experiment included six plants per treatment with three replications (total 18 plants) and pots were set-up in a randomized design. Plants were grown in a growth chamber under the conditions described above.

### Statistical Analyses

The data were subjected to one-way analysis of variance (ANOVA) in the software package SPSS-22 statistical software (SPSS, Inc., Chicago, IL, USA). Mean comparisons were conducted by the least significant difference (LSD) (*P* = 0.05) test.

## Results

### The Antimicrobial Activity of Plant Extracts

The inhibitory effect of extracts from *Z. capitata* and *H. perforatum*, which were tested against diverse enteric pathogens (*A. baumanii* 60649, *K. oxytoca* 6653, *K. pneumoniae* 40602, *K. aerogenes* NCTC 8172, *C. freundii* 82073, *S. aureus* MRSA 16, *E. faecalis* NCTC 775, *Proteus rettgeri* NCIMB 9570, *P. aeruginosa* NCTC 6749, and *E. coli* NCTC 9001) at a concentration of 10 mg ml^-1^, resulted in different extents of inhibition (**Table 1**). The strains *A. baumanii* 60649, *E. coli* NCTC 9001, *E. faecalis* NCTC 775, *K. oxytoca* 6653, *K. pneumoniae* 40602, *P. aeruginosa* NCTC 6749, and *S. aureus* MRSA 16 were inhibited by the extract of *H. perforatum*. However, extract of *Z. capitata* did not exhibit any potential antibacterial activity against the 10 tested pathogens. Extracts of *H. perforatum* exhibited potential antifungal activity against *F. oxysporum* and *A. alternata*, whereas the extract of *Z. capitata* did not exhibit any inhibitory activity against the tested fungal strains.

**Table 1:** Antimicrobial activity of extracts obtained from *Hypericum perforatum* and *Ziziphora capitata*^a^.

Plant species	*A. baumanii*	*C. freundii*	*E. coli*	*E. faecalis*	*K. oxytoca*	*K. pneumoniae*	*K. aerogenes*	*P. rettgeri*	*P. aeruginosa*	*S. aureus*	*F. solani*	*F. oxysporum*	*A. alternata*
*H. perforatum*	+	-	+	+	+	+	-	-	+	+	-	+	+
*Z. capitata*	-	-	-	-	-	-	-	-	-	-	-	-	-


^a^Disk diffusion method: (+) susceptibility > 2 mm, (-) absence of susceptibility.

### Enumeration, Isolation, and Identification of Endophytic Bacteria

The total number of endophytic bacterial isolates in the root tissue of *Z. capitata* was significantly higher (4.5 ± 0.8 × 10^3^ CFU g^-1^ of fresh root tissue) than in *H. perforatum* roots (2.6 ± 0.71 × 10^3^ CFU g^-1^ of fresh root tissue). Isolates were chosen randomly from the dilution plates exhibiting different colonial morphology, forms, texture, and color from each plate. A total of 18 bacterial isolates were derived from *H. perforatum* and 15 isolates from *Z. capitata*. Taxonomic investigation by MALDI-TOF MS revealed that the majority of strains were identified with secure genus identification and probable species identification (**Table 2**). The endophytes from the root of *H. perforatum* were affiliated with nine genera, whereas 14 isolates were identified at the species level. *Achromobacter* was the predominant genus, which was followed by the genus *Pseudomonas*. Furthermore, isolates affiliated with the genera *Arthrobacter, Bacillus, Erwinia, Pantoea, Serratia*, and *Stenotrophomonas* were found. The most abundant species was *Achromobacter piechaudii* (S22a, S7a, S7) (**Table 2**). A total of five bacterial genera were isolated from the root of *Z. capitata* (**Table 2**). The most abundant isolates of *Z. capitata* were also identified as *A. piechaudii* (M11, M6, M31, M24, M41). Members of the genera *Serratia, Stenotrophomonas*, and *Erwinia* were not identified among the endophytes from *Z. capitata*.

**Table 2:** Matrix-assisted laser desorption ionization (MALDI) biotyper-based identification of culturable endophytic bacteria isolated from the root of *Hypericum perforatum* and *Ziziphora capitata*, and traits related to biocontrol and/or plant growth-promoting activity of bacterial strains.

Plant	Isolate	Identity according to MALDI-TOF MS	Score^∗^	Cellulase	Protease	β-1,3-glucanase	HCN	IAA (μg/ml)	
								Tr–	Tr+
	S1	*Arthrobacter crystallopoietes*	++	+	+	**-**	+	19.8	22.1
*Hypericum perforatum*	S22a	*Achromobacter piechaudii*	+++	**-**	**-**	**-**	**-**	7.3	7.5
	S7a	*Achromobacter piechaudii*	++	+	+	+	+	6.4	8
	S7	*Achromobacter piechaudii*	++	**-**	+	**-**	**-**	17.5	17.1
	S23	*Achromobacter spanius*	++	**-**	**-**	**-**	**-**	3.8	4.7
	S14	*Achromobacter* sp.	+	+	+	+	+	15.2	19.6
	S2	*Bacillus* sp.	+	**-**	**-**	**-**	**-**	4.2	15.5
	S40	*^#^Bacillus cereus*	++	**-**	+	**-**	+	12.2	14.8
	S4a	*Enterobacter cloacae*	++	+	+	+	**-**	4.9	5.1
	S4	*Erwinia persicina*	++	+	+	+	+	9	8.5
	S19	*Pseudomonas koreensis*	++	**-**	+	**-**	**-**	10.7	10.3
	S5	*Pseudomonas putida*	++	**-**	**-**	**-**	**-**	9.4	11.2
	S25	*Pseudomonas thivervalensis*	+	**-**	+	**-**	**-**	6.2	18.4
	S24	*Pseudomonas pseudoalcaligenes*	++	**-**	+	**-**	**-**	4.3	4.8
	S3	*Pseudomonas kilonensis*	++	**-**	+	**-**	**-**	3.9	4.5
	S22	*Pantoea agglomerans*	++	**-**	+	**-**	**-**	12.3	55
	S26	*Serratia liquefaciens*	+++	**-**	+	**-**	+	8.7	15
	S9	*Stenotrophomonas* sp.	+	+	+	+	+	8	16.3
*Ziziphora capitata*	M11	*Achromobacter piechaudii*	++	**-**	**-**	**-**	**-**	2.8	2.9
	M6	*Achromobacter piechaudii*	++	**-**	**-**	**-**	**-**	4.3	4.3
	M31	*Achromobacter piechaudii*	++	**-**	**-**	**-**	**-**	3.5	3.2
	M24	*Achromobacter piechaudii*	++	**-**	**-**	**-**	**-**	3.9	3.7
	M41	*Achromobacter piechaudii*	++	**-**	+	**-**	**-**	9.1	9.6
	M19	*Achromobacter* sp.	+	**-**	+	**-**	**-**	5.7	5.5
	M18	*Achromobacter spanius*	++	+	**-**	+	**-**	4.1	4.3
	M8	*Achromobacter spanius*	++	+	+	**-**	**-**	3.1	3.6
	M9a	*Bacillus altitudinis*	++	**-**	**-**	**-**	**-**	9.5	49.9
	M14	*^#^Bacillus cereus*	++	**-**	**-**	**-**	**-**	4.1	4.4
	M20	*Enterobacter cloacae*	+++	**-**	+	**-**	**-**	12.2	15
	M17	*Enterobacter* sp.	+	**-**	**-**	**-**	**-**	5.6	5.3
	M13	*Pantoea agglomerans*	+++	**-**	**-**	**-**	**-**	16.1	52.5
	M6a	*Pseudomonas kilonensis*	+	**-**	**-**	**-**	**-**	6	7.3
	M2	*Pseudomonas thivervalensis*	++	**-**	**-**	**-**	**-**	2.6	2.8


^∗^++, score value 2.300–3.000, highly probable species identification; +, score value 2.000–2.299, secure genus identification and probable species identification; +, score value 1.700–1.999, probable genus identification; ^#^*Bacillus anthracis*, *B. cereus*, *B. mycoides*, *B. pseudomycoides*, *B. thuringiensis* and *B. weihenstephanensis* are closely related and members of the *Bacillus cereus* group, which cannot be differentiated by MALDI-TOF MS.

### Beneficial Plant Traits of Endophytic Bacteria

All endophytes isolated from *H. perforatum* and *Z. capitata* were screened for multiple plant growth-promoting traits. Most of the bacterial isolates exhibited one or more plant growth-promoting activities. The production of phytohormone IAA by bacterial isolates is presented in **Table 2**. The highest level of IAA production was observed for *Arthrobacter crystallopoietes* S1 (19.8 μg ml^-1^), *A. piechaudii* S7 (17.5 μg ml^-1^), *Achromobacter* sp. S14 (15.2 μg ml^-1^), *Pantoea agglomerans* S22 (12.3 μg ml^-1^), and *Bacillus cereus* S40 (12.2 μg ml^-1^), which were isolated from *H. perforatum.* Two isolates, *Enterobacter cloacae* M20 and *P. agglomerans* M13, isolated from *Z. capitata* also exhibited high IAA production in culture media (12.2 and 16.1 μg ml^-1^, respectively). The presence of tryptophan did not stimulate auxin production in the majority of the isolates, whereas only four isolates from *Z. capitata* revealed an increase in IAA synthesis: *Bacillus* sp. S2 (15.5 μg ml^-1^), *P. agglomerans* S22 (μg ml^-1^), *Serratia liquefaciens* S26 (15.0 μg ml^-1^), *Stenotrophomonas* sp. S9 (16.3 μg ml^-1^) and two isolates, *B. altitudinis* M9a (49.9 μg ml^-1^) and *P. agglomerans* M13 (52.5 μg ml^-1^) from *H. perforatum* (**Table 2**). All isolates isolated from *H. perforatum*, except *Achromobacter spanius* S23, *Bacillus* sp. S2 and isolate S9, were able to produce one or more cell wall-degrading enzymes. In contrast, only four isolates from *Z. capitata* (*A. piechaudii* M41*, Achromobacter* sp. M19, *E. cloacae* M20 and isolate M8) were able to produce proteases, and only one isolate, *A. spanius* M18, produced cellulase and β-1,3-glucanase. HCN was not produced by any isolate from *Z. capitata*, whereas seven isolates isolated from *H. perforatum* were able to produce HCN (**Table 2**).

**FIGURE 1:**
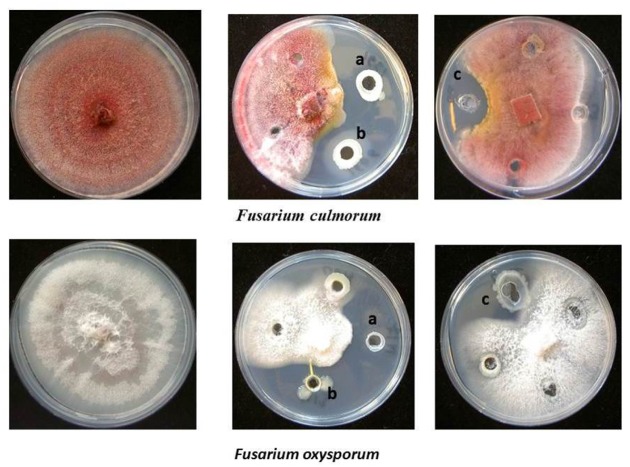
**Antagonistic activity of endophytic bacterial isolates. (a)** *Pseudomonas pseudoalcaligenes* S24, **(b)** *Stenotrophomonas* sp. S9 isolated from *Hypericum perforatum*, and **(c)** *Enterobacter* sp. M17 isolated from *Ziziphora capitata* against *Fusarium culmorum* and *Fusarium oxysporum.*

Antagonistic activity was recorded for endophytes against plant pathogenic fungi *F. oxysporum* f. sp. *radicis-lycopersici*, *F. solani*, *F. culmorum, G. graminis* pv. *tritici*, *A. alternata*, and *B. cinerea* and the oomycete *P. ultimum*. As presented in **Table 3**, all isolates from *H. perforatum* exhibited antagonistic behavior to one or more of the tested plant pathogenic fungi. The isolates *A. crystallopoietes* S1, *A. piechaudii* S7, *Pseudomonas koreensis* S25, *Pseudomonas pseudoalcaligenes* S24, and *Stenotrophomonas* sp. S9 were highly effective against six fungal pathogens and exhibited the highest inhibition of mycelial growth (**Figure 1**). Among the isolates from *Z. capitata*, only *P. agglomerans* M13 exhibited antagonistic activity against five fungal pathogens, but the *in vitro* inhibition of the mycelium was lower than that of the other isolates. In general, *H. perforatum*, which exhibited a broad spectrum of antimicrobial activity, supported a higher proportion of antagonistic endophytes compared with *Z. capitata*.

**Table 3:** Antagonistic activity of culturable endophytic bacterial isolates associated with *Hypericum perforatum* and *Ziziphora capitata* against soil-borne fungal pathogens.

Plant	Isolate	Bacterial isolates	*F. oxysporum*	*B. cinera*	*P. ultimum*	*F. culmorum*	*F. solani*	*G. graminis*	*A. alternata*
*Hypericum perforatum*	S1	*Arthrobacter crystallopoietes*	+	+	+	+	+	+	+
	S22a	*Achromobacter piechaudii*	+	**-**	**-**	+	+	**-**	**-**
	S7a	*Achromobacter piechaudii*	+	**-**	**-**	+	+	+	+
	S7	*Achromobacter piechaudii*	+	+	+	+	+	+	+
	S23	*Achromobacter spanius*	+	**-**	**-**	+	+	+	+
	S14	*Achromobacter* sp.	**-**	**-**	**-**	+	**-**	+	+
	S2	*Bacillus* sp.	+	**-**	+	+	+	+	**-**
	S40	*Bacillus cereus*	+	+	**-**	+	+	+	+
	S4a	*Enterobacter cloacae*	+	**-**	+	+	+	**-**	+
	S4	*Erwinia persicina*	+	**-**	**-**	+	+	+	+
	S19	*Pseudomonas koreensis*	+	**-**	+	+	+	+	+
	S5	*Pseudomonas putida*	+	**-**	+	+	+	+	+
	S25	*Pseudomonas thivervalensis*	+	**-**	+	+	+	+	+
	S24	*Pseudomonas pseudoalcaligenes*	+	+	+	+	+	+	+
	S3	*Pseudomonas kilonensis*	+	**-**	**-**	+	**-**	+	+
	S22	*Pantoea agglomerans*	+	**-**	**-**	+	**-**	+	**-**
	S26	*Serratia liquefaciens*	+	**-**	+	+	**-**	+	+
	S9	*Stenotrophomonas* sp.	+	+	**-**	+	+	+	+
*Ziziphora capitata*	M11	*Achromobacter piechaudii*	**-**	**-**	**-**	**-**	**-**	**-**	+
	M6	*Achromobacter piechaudii*	**-**	**-**	**-**	**-**	+	**-**	**-**
	M31	*Achromobacter piechaudii*	**-**	+	**-**	**-**	**-**	**-**	**-**
	M24	*Achromobacter piechaudii*	**-**	**-**	**-**	**-**	**-**	**-**	**-**
	M41	*Achromobacter piechaudii*	**-**	**-**	**-**	**-**	**-**	**-**	**-**
	M19	*Achromobacter* sp.	**-**	**-**	**-**	**-**	+	**-**	**-**
	M18	*Achromobacter spanius*	**-**	**-**	**-**	**-**	**-**	**-**	**-**
	M8	*Achromobacter spanius*	**-**	**-**	+	+	+	+	**-**
	M9a	*Bacillus altitudinis*	**-**	**-**	**-**	**-**	**-**	**-**	**-**
	M14	*Bacillus cereus*	**-**	**-**	**-**	**-**	**-**	**-**	**-**
	M20	*Enterobacter cloacae*	**-**	**-**	**-**	**-**	**-**	**-**	**-**
	M17	*Enterobacter* sp.	+	**-**	**-**	**-**	**-**	**-**	**-**
	M13	*Pantoea agglomerans*	+	**-**	**-**	+	+	**-**	**-**
	M6a	*Pseudomonas kilonensis*	+	**-**	**-**	+	+	+	+
	M2	*Pseudomonas thivervalensis*	**-**	**-**	**-**	**-**	**-**	**-**	**-**


+ Presence of antagonism; - absence of antagonism.

### Biological Control and Plant Growth Promotion

**FIGURE 2:**
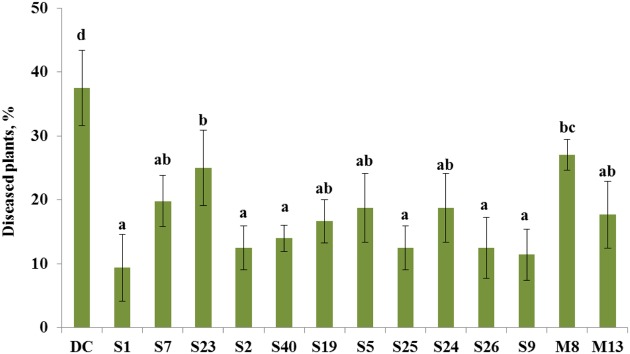
**Control of tomato foot and root rot caused by *F. oxysporum* by selected antagonistic endophytic bacteria (*Arthrobacter crystallopoietes* S1, *Achromobacter piechaudii* S7, *Achromobacter spanius* S23, *Bacillus* sp.** S2, *Bacillus cereus* S40, *Pseudomonas putida* S19, *Pseudomonas thivervalensis* S5, *Pseudomonas koreensis* S25, *P. pseudoalcaligenes* S24, *Serratia liquefaciens* S26, *Stenotrophomonas* sp. S9, *A. spanius* M8, *Pantoea agglomerans* M13). DC- disease control (soil infested with *F. oxysporum* spores), healthy control (no *F. oxysporum* spores added to the soil) had only 2% diseased plants. Column means marked by different letters indicate significant differences based on Turkey’s HSD test at *P* < 0.05.

The bacterial isolates that exhibited antagonistic activity against a wide range of fungal pathogens *in vitro* were selected to evaluate their ability to suppress tomato foot and root rot caused by *F. oxysporum* f. sp. *radicis-lycopersici* in a pot experiment. In non-infested soil, the portion of diseased plants was 2%, whereas in the presence of the pathogen, the portion of plants that exhibited disease symptoms increased to 38% (**Figure 2**). The selected antagonistic bacterial isolates *A. crystallopoietes* S1, *Bacillus* sp. S2, *B. cereus* S40, *P. koreensis* S25, *S. liquefaciens* S26, and *Stenotrophomonas* sp. S9, exhibited a statistically significant (*P* < 0.05) disease reduction (up to 9%) compared with *Fusarium*-infected control plants (**Figure 2**). Several isolates, namely *A. piechaudii* S7, *Pseudomonas putida* S19, *Pseudomonas thivervalensis* S5, *P. pseudoalcaligenes* S24, and *P. agglomerans* M13 reduced disease incident, but the effects were not significant.

**FIGURE 3:**
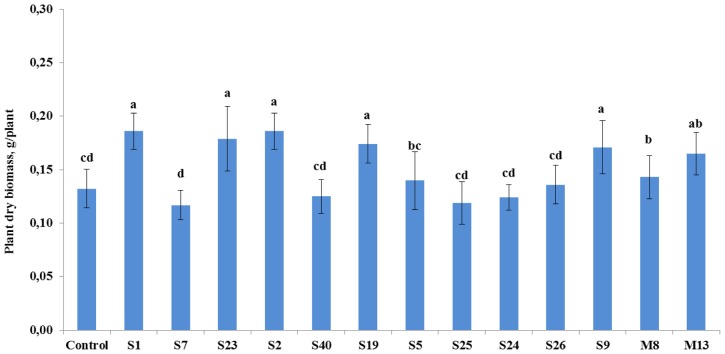
**The effect of seedling inoculation with selected antagonist endophytic isolates on the dry weight of tomato (*A. crystallopoietes* S1, *A. piechaudii* S7, *A. spanius* S23, *Bacillus* sp.** S2, *B. cereus* S40, *P. putida* S19, *P. thivervalensis* S5, *P. koreensis* S25, *P. pseudoalcaligenes* S24, *S. liquefaciens* S26, *Stenotrophomonas* sp. S9, *A. spanius* M8, *P. agglomerans* M13). Column means marked by different letters indicate significant differences based on Turkey’s HSD test at *P* < 0.05.

The antagonistic endophytic bacterial isolates were also effective on the growth of tomato plants under controlled conditions (**Figure 3**). Statistical analysis showed that growth stimulatory effects of the isolates *A. crystallopoietes* S1, *A. spanius* S23, *Bacillus* sp. S2, *P. putida* S19, and *Stenotrophomonas* sp. S9 increased plant biomass significantly (*P* < 0.05) between 30 and 41%. However, four strains namely *A. piechaudii* S7, *B. cereus* S40, *P. koreensis* S25, and *P. pseudoalcaligenes* S24 reduced plant growth response, leading to a decrease in plant dry biomass between 5.3 and 11.4% (**Figure 3**).

## Discussion

In our study, we analyzed the antimicrobial activity of plant extracts of *H. perforatum* and *Z. capitata*, and characterized plant beneficial traits of their associated culturable endophytic bacteria. Both parameters exhibited a relationship – *Hypericum* plant extracts exhibited greater antimicrobial activity and harbored a higher abundance of endophytes with antagonistic activity than *Ziziphora*, which lacks antimicrobial activity. In detail, *H. perforatum* was proved to possess potential antimicrobial activity against a wide range of pathogenic bacteria (*A. baumanii*, *E. coli, E. faecalis, K. oxytoca, K. pneumoniae, P. aeruginosa*, *S. aureus*) as well as fungi (*F. oxysporum, A. alternata*), whereas the extract of *Z. capitata* did not exhibit any inhibitory activity against the tested microbes. A similar observation for *H. perforatum* was reported by Maleš et al. (2006), who found that methanol extracts exhibited strong antibacterial activity against *S. aureus*, *S. epidermidis*, *E. faecalis*, and *Bacillus subtilis*.

In our study, we observed a lower number of endophytes in *H. perforatum* compared to *Z. capitata* that exhibited antibacterial activity. This is consistent with the report of Ahmed et al. (2014), who also reported a smaller microbial population in the rhizosphere of *M. chamomilla*, which possesses abundant antibacterial activity against pathogenic bacteria (Munir et al., 2014). Our findings suggest that host plants differing in their antibacterial activity exhibited selective effects on physiological properties of endophytes. The understanding of interactions of endophytic bacteria with host plants includes the production of phytohormones, siderophores, and antifungal compounds, which have been well-documented previously by various authors (Berg et al., 2013, 2014; Cho et al., 2015; Egamberdieva et al., 2015a,b). Endophytic bacteria can also improve plant growth by protecting plants against soil-borne diseases or various environmental stresses (Berg et al., 2014; Cao et al., 2014). We have observed that endophytic bacteria associated with both investigated plants exhibited multiple plant beneficial activities, such as the production of IAA, HCN and cell-wall-degrading enzymes. Moreover, the endophytic bacteria associated with *H. perforatum* demonstrated higher antagonistic activity as compared with endophytes of *Z. capitata*. This observation is consistent with Gorluk et al. (2009), who also reported a higher proportion of antagonistic endophytes associated with *Chelidonium majus* L., which is known for its antimicrobial potential (Zuo et al., 2008; Baker and Satish, 2013) against fungal pathogens. Furthermore, it is has been documented that endophytic microbes associated with medicinal plants may produce the same metabolites as their hosts and have been considered a potential source of biologically active metabolites (Mehanni and Safwat, 2010). For example, endophytic species (e.g., *Pseudomonas*, *Bacillus*) associated with *Aloe vera* exhibit antibacterial activity against human pathogenic bacteria, such as *S. aureus*, *Streptococcus pyogenes*, *P. aeruginosa*, and *E. coli* (Nejatzadeh-Barandoz, 2013), and produce bioactive compounds with antimicrobial activities (Akinsanya et al., 2015).

In our study, endophytic isolates which exhibited antagonistic activity against a wide range of fungal pathogens were evaluated for their capability to suppress tomato foot and root rot caused by *F. oxysporum.* All selected bacterial isolates of *A. crystallopoietes* S1, *Bacillus* sp. S2, *B. cereus* S40, *P. koreensis* S25, *S. liquefaciens* S26, and *Stenotrophomonas* sp. S9, exhibited statistically significant disease reduction compared with the *Fusarium*-infected control plants. These observations demonstrate the capability of endophytes to protect plants from soil-borne diseases. In accordance with these results, there is a report of the biological control of *Verticillium* wilt disease of cotton by endophytic bacteria *B. subtilis* KDRE 01 and *B. megaterium* KDRE 25, isolated from the medical plant *Sophora alopecuroides* (Lin et al., 2013). It has been also reported that *Stenotrophomonas maltophilia* which is an antagonist against *Ralstonia solanacearum* significantly suppressed potato brown rot in Egyptian clay soil (Messiha et al., 2007). Moreover, five isolates namely *A. crystallopoietes* S1, *A. spanius* S23, *Bacillus* sp. S2, *P. putida* S19, and *Stenotrophomonas* sp. S9 with antifungal activity exhibited enhancement of tomato growth. This finding is consistent with Wei et al. (2014), who also observed an enhanced growth of tomato plants by *B. subtilis* isolated from the rhizosphere of the traditional Chinese medicinal herb *Trichosanthes kirilowii*. In another study, endophytic bacteria isolated from a common weed *Cassia occidentalis* used in several traditional medicines, were able to produce IAA and stimulated growth of mung bean in pot experiments (Arun et al., 2012).

## Conclusion

The results from our pilot study of ongoing research provide insights about plant beneficial traits of culturable endophytic bacteria associated with the medicinal plants *H. perforatum* and *Z. capitata* with contrasting antimicrobial activities. We observed that *H. perforatum* with antibacterial activity supported more bacteria with antagonistic activity, as compared to *Z. capitata*. The antagonistic isolates were able to control tomato root rot caused by *F. oxysporum* under greenhouse conditions and could be a cost effective source for agro-based biological control agents. However, these findings indicate that further research is necessary to resolve the impact of medicinal plant species with contrasting antimicrobial activity on the endophytic microbial community in more detail, and to identify biological active compounds produced by the hosts and their endophytes.

## Author Contributions

DE, SW, and GB did experimental design work. DE and UB conducted experiments. PA analyzed the data. DE, SW, UB, and GB wrote the manuscript. All authors read and approved the Manuscript.

## Conflict of Interest Statement

The authors declare that the research was conducted in the absence of any commercial or financial relationships that could be construed as a potential conflict of interest. The reviewer SPG and handling Editor declared their shared affiliation, and the reviewer SPG declared a past co-authorship with one of the authors UB to the handling Editor, who ensured that the process met the standards of a fair and objective review.
